# Non-verbal emotion communication training induces specific changes in brain function and structure

**DOI:** 10.3389/fnhum.2013.00648

**Published:** 2013-10-17

**Authors:** Benjamin Kreifelts, Heike Jacob, Carolin Brück, Michael Erb, Thomas Ethofer, Dirk Wildgruber

**Affiliations:** ^1^Department of Psychiatry and Psychotherapy, Eberhard Karls University of TübingenTübingen, Germany; ^2^Department of Biomedical Magnetic Resonance, Eberhard Karls University of TübingenTübingen, Germany

**Keywords:** fMRI, VBM, superior temporal sulcus, fusiform face area, neuroticism, emotion sensitivity

## Abstract

The perception of emotional cues from voice and face is essential for social interaction. However, this process is altered in various psychiatric conditions along with impaired social functioning. Emotion communication trainings have been demonstrated to improve social interaction in healthy individuals and to reduce emotional communication deficits in psychiatric patients. Here, we investigated the impact of a non-verbal emotion communication training (NECT) on cerebral activation and brain structure in a controlled and combined functional magnetic resonance imaging (fMRI) and voxel-based morphometry study. NECT-specific reductions in brain activity occurred in a distributed set of brain regions including face and voice processing regions as well as emotion processing- and motor-related regions presumably reflecting training-induced familiarization with the evaluation of face/voice stimuli. Training-induced changes in non-verbal emotion sensitivity at the behavioral level and the respective cerebral activation patterns were correlated in the face-selective cortical areas in the posterior superior temporal sulcus and fusiform gyrus for valence ratings and in the temporal pole, lateral prefrontal cortex and midbrain/thalamus for the response times. A NECT-induced increase in gray matter (GM) volume was observed in the fusiform face area. Thus, NECT induces both functional and structural plasticity in the face processing system as well as functional plasticity in the emotion perception and evaluation system. We propose that functional alterations are presumably related to changes in sensory tuning in the decoding of emotional expressions. Taken together, these findings highlight that the present experimental design may serve as a valuable tool to investigate the altered behavioral and neuronal processing of emotional cues in psychiatric disorders as well as the impact of therapeutic interventions on brain function and structure.

## Introduction

Perception and correct interpretation of non-verbal emotional cues from voice and face is essential for intact social interaction and social functioning. Typically, this ability is acquired during childhood and youth in a rather implicit manner as a part of our upbringing. Nevertheless, these perceptive skills can be trained explicitly. It has been shown that such training in healthy individuals improves both their confidence and their decoding performance (Constanzo, 1992), their social and interpersonal skills (Matsumoto and Hwang, 2011) and heightens their non-verbal perceptiveness and sensitivity (Klinzing and Jackson, 1987).

The processing of non-verbal emotional signals has been found to be altered in different psychiatric conditions including depression, anxiety disorders, bipolar disorder, schizophrenia, psychopathy, and borderline personality disorder (e.g., Domes et al., 2009; Demenescu et al., 2010; Kohler et al., 2010, 2011; Dawel et al., 2012; Samame et al., 2012). Consequentially, emotion perception trainings may also appear as a means to reduce this deficit and improve social communication and functioning in psychiatric patients. In this regard, structured behavioral trainings have been found to be effective in ameliorating facial emotion recognition and social functioning in schizophrenia (Kurtz and Richardson, 2012). However, much less is known about the neural bases of such emotional communication trainings, both in healthy individuals and psychiatric patients. While a recent functional magnetic resonance imaging (fMRI) study in schizophrenic patients described alterations in cerebral activation patterns following a facial affect recognition training (Habel et al., 2010), in healthy individuals, to our knowledge, no study describing the neuronal effects of an emotion communication training has been published to date.

Using fMRI in healthy individuals, it was demonstrated that the processing of non-verbal emotional facial and vocal cues (i.e., facial expressions and tone of voice) is associated with increased activation of sensory cortices specialized for the processing of human (emotional) voices (e.g., Belin et al., 2000; Wildgruber et al., 2006; Ethofer et al., 2012) and faces (e.g., Kanwisher et al., 1997; Posamentier and Abdi, 2003), and limbic brain areas (e.g., Posamentier and Abdi, 2003; Wildgruber et al., 2006; Brück et al., 2011b). The combined perception and integration of these signals is associated with a further increase in activation in the posterior temporal sulcus (pSTS), thalamus, the face processing area in the fusiform gyrus (FFA) and the amygdala (e.g., Pourtois et al., 2005; Kreifelts et al., 2007, 2009, 2010; Robins et al., 2009; Klasen et al., 2011, for a review see Klasen et al., 2012). Irrespective of the sensory modality of non-verbal cue presentation, lateral prefrontal and supplementary motor cortices are engaged in the evaluation of these signals and response selection (e.g., Schirmer and Kotz, 2006; Brück et al., 2011b; Ethofer et al., 2013).

In the present study, it was our goal to determine the effects of a non-verbal emotional communication training (NECT) on the neuronal processing of non-verbal cues from voice and face in healthy individuals as a feasibility study for further investigations in patients with psychiatric conditions.

To this end, healthy participants either received a four-week NECT or an equally intensive but essentially non-communicative training in Sudoku as control condition. The NECT took place as group training with the central training elements revolving around a board game complemented by non-verbal exercises. Sudoku, in contrast, is a popular Japanese pastime in form of number riddles to be solved by pure logic. Before and after the training interval, the participants took part in three fMRI experiments: During the first experiment the participants had to judge the valence of short video sequences portraying faces speaking short sentence with varying (happy, neutral, angry) vocal and facial expressions. Valence ratings and cerebral responses were treated as outcome variables. The additional two fMRI experiments were standard localizer experiments to identify voice- and face-selective brain regions.

First, behavioral and cerebral data were analyzed within the framework of an analysis of variance (ANOVA) with the factors time point [before (T0) vs. after (T1) training], training type (NECT vs. SUDOKU) and non-verbal emotion (emotional vs. neutral cues). Both general and emotion-specific effects were assessed.

With regard to the training outcome, we expected that cerebral effects of NECT would occur foremost in those brain areas involved in the processing of non-verbal emotional signals as well as in face- and voice-selective cortices.

Secondly, as it has been shown that certain personality factors such as neuroticism, on the one hand, influence the processing of non-verbal emotional signals (e.g., Stein et al., 2007; Cremers et al., 2010; Suslow et al., 2010; Brück et al., 2011a; Kehoe et al., 2012), and on the other hand, are associated with an increased risk to develop a psychiatric condition (e.g., the association of neuroticism and depression and anxiety; Brandes and Bienvenu, 2006; Klein et al., 2011), we investigated if personality factors modulate the behavioral or cerebral effects of NECT. To this end, the NEO-Five Factor Inventory (NEO-FFI; Borkenau and Ostendorf, 2008) capturing the personality factors neuroticism, extraversion, openness to experience, agreeableness and conscientiousness was completed by all participants. The individual personality ratings were then used as explanatory covariates for observed NECT effects.

Thirdly, NECT-associated changes in the behavioral and cerebral sensitivity to non-verbal emotional cues were correlated to identify behaviorally relevant cerebral correlates of NECT.

Finally, cerebral correlates of training have not only been observed on the functional but also on the structural level (e.g., Draganski et al., 2004). Therefore, we further tested if NECT increased gray matter (GM) volume in face- and voice-selective cortices and other brain regions with NECT-induced changes in their neuronal responses.

## Materials and methods

### Participants

Sixteen healthy and right-handed (Edinburgh Handedness Inventory; Oldfield, 1971) individuals were initially included into the study. None of the participants reported any current or past substance abuse problems, neurological or psychiatric illnesses, nor indicated any hearing difficulties or uncorrected vision impairments. Moreover, none of the participants reported to be taking any medication. Of the 16 participants, eight were randomized to the NECT group [4 females; mean age = 24.88 years, standard deviation (SD) = ± 1.89 years] and eight were randomized to the SUDOKU training group. Of these eight, two participants had to be excluded from the study (i.e., one participant did not attend the training and one had to be excluded due to a structural cerebral anomaly). Thus, six individuals remained in the Sudoku training group (2 females; mean age = 25.33 years, *SD* = ± 1.51 years) and 14 individuals (6 females; mean age = 25.07 years, *SD* = ±1.69 years) were included in the analyses.

At the beginning of the study all participants completed the German version of the NEO-FFI (Borkenau and Ostendorf, 2008) based on the works of Costa and McCrae. The NEO-FFI is a multidimensional self-report personality inventory with 60 items which assesses the following five personality factors: neuroticism (N), extraversion (E), openness (O) to experience, agreeableness (A), and conscientiousness (C). The NECT group scored as follows (mean ± *SD*): N: 1.21 ± 0.33; E: 2.57 ± 0.31; O: 2.68 ± 0.22; A: 2.55 ± 0.71; C: 2.47 ± 0.5. The results of the SUDOKU group were: N: 1.74 ± 0.71; E: 2.33 ± 0.43; O: 2.82 ± 0.39; A: 2.67 ± 0.4; C: 2.44 ± 0.32.

### Ethics statement

The study was performed according to the principles of the Code of Ethics of the World Medical Association (Declaration of Helsinki), and with the approval of the ethics review board of the University of Tübingen. Before inclusion in the study, all participants gave written informed consent. The participants were monetarily compensated for the study participation.

### Stimulus material, tasks, and procedure

The stimulus material comprised a set of 120 video films of 10 professional actors (5 females) speaking short sentences. The stimuli contained verbal (i.e., six short German sentences with self-referential content) as well as non-verbal information (i.e., facial expression and tone of voice) about the emotional states of the respective speakers. The non-verbal expressions differed in their valence: One third (40) of the video sequences portrayed an angry expression, one third a happy expression and one third an emotionally neutral expression. The stimulus material was balanced with respect to the valence of verbal stimulus content which included negative, positive and neutral sentences. The stimuli had a mean duration of 1459 ms (*SD* = 317 ms). For further details on stimulus production, editing, validation and selection please refer to Jacob et al. (2013).

The software Presentation (Neurobehavioral Systems Inc., Albany, CA, USA) was used for the experiment. Video films were back-projected onto a screen in the scanner bore about 50 cm behind the participant's head. The participants viewed the screen through a mirror system mounted on the head coil. Magnetic resonance compatible headphones (Sennheiser electronic GmbH & Co. KG, Wedemark-Wennebostel, Germany; in-house modified) were used for sound transmission.

The 120 video films used for the study were divided into two equal blocks balanced for non-verbal and verbal stimulus valence as well as the gender of the speaker. The order of stimulus presentation was randomized within blocks, and the sequence of the two stimulus blocks was balanced across participants. In each block 10 null events each with a duration of 10 s were randomly inserted in the stimulus sequence. The stimulus blocks were presented within separate imaging runs. Stimulus onset was jittered relative to scan onset in steps of 1/4 repetition time (TR). It was the participant's task to judge the emotional state of the speaker on a 4-point valence scale (“–” = highly negative, “−” = negative, “+” = positive, “++” = highly positive) as precisely and as fast as possible. A horizontally flipped scale was used for half of the participants. The participants' responses were transmitted through button presses on a four-buttons fiber optic response pad (LUMItouch, Photon Control Inc., Burnaby, BC, Canada). The response window was set to 5 s in duration and was time-locked to the onset of the videos. After the offset of the videos, the 4-point valence scale was presented. The participants were acquainted with the experimental setting through a short training session outside the scanner. The stimuli employed in the training session were not part of the stimulus set used in the main experiment.

After the main experiment two standard functional localizer experiments were run to identify face- and voice-selective brain regions. The face localizer was adapted from previous studies on face processing (Kanwisher et al., 1997; Epstein et al., 1999) and included pictures from four different categories (faces, houses, objects, and natural scenes) presented using a block-design. The voice localizer data were acquired using a block design experiment validated in previous research (Belin et al., 2000; Kreifelts et al., 2009). The employed stimuli included human voices (e.g., speech, sighs, laughs), animal sounds (cries of various animals), and environmental sounds (e.g., doors, telephones, cars). For details on the localizer experiments see Kreifelts et al. (2010).

All participants took part in the fMRI experiments once before the training (T0) and once after training (T1). Randomization to the different types of training took place directly after the first measurement session before analyzing any data.

### Non-verbal emotion communication training (NECT)

NECT is a four-week group training program (18 days, 1 h per day) consisting of a game, theoretical discussions and supplementary exercises held in a group setting with eight participants.

#### Non-verbal communication game

The central part of the training was a non-verbal communication game in the form of a board game. Playing the game involves extensive practice expressing emotions as well as understanding emotions as the players take turns expressing emotions and perceiving emotions in the other players. The basic rules can be summarized as follows: Sentence cards (i.e., cards, each displaying a single sentence) are put face down on their allotted space on the board. Emotion cards (i.e., cards, each displaying an emotion label) are placed in the middle of the board. Six Emotion cards are then put face up on their allotted spaces on the board numbered from 1 to 6. Each player team chooses one of six tokens to represent their position on the board. Each team also receives a set of Number cards ranging from 1 to 6. The team that was chosen to start draws one of their six Number cards referring to one of the six Emotion cards. Each of the two players within a team has to draw a Sentence card and read the sentence in the respective emotional tone of voice and with an appropriate facial expression. The other teams have to guess which emotion was conveyed by the tone of voice and the facial expression. In the first round, each team is allowed to discuss their decision in a non-verbal manner (i.e., by pointing to the respective number cards referring to the different emotions). Once all quiz teams have come to a decision, they show their cards simultaneously. In case of a correct answer, the respective quiz team is allowed to move their token one step in the direction of the goal. The team which performed the emotional expressions is allowed to move their token in the direction of the goal for the number of spaces indicated by the number of correct answers by the other teams based on their performance. In the second round, the procedure is similar, but each team is given two sets of Number cards, one for each team player. Now, each quiz team is allowed to silently discuss their decision, however not by pointing to the cards but by using means of non-verbal communication (e.g., by using facial expressions or gestures of the respective emotion). The quiz teams are allowed to move their token in the direction of the goal one step in case of correct AND matching answers. In the third round, the quiz teams are not allowed to communicate within the teams. Each team player has to make her/his own decision. The quiz team is allowed to move their token in the direction of the goal one step in case of correct AND matching answers. To diversify the game and increase its training effect, some modifications were made during the course of the training (e.g., to avoid imitations and to broaden the range of expressions, one of the two acting players had to wait outside the room until his/her teammate has finished his/her performance).

#### Non-verbal communication—supplementary exercises

The supplementary exercises were based on a playful approach to improve non-verbal communication skills. Exercises were provided by a book written by Funcke (2006) describing different exercises training the ability to express and perceive non-verbal emotional signals. The following exercises were part of the training:

- Körpersprachespiel (i.e., game of body language; pages 203–204)
- Der Ton macht die Musik (i.e., it is not what you say, it is the way you say it; pages 139–140)
- Mimische Kette (i.e., chain of facial expressions; pages 169–170)
- Audienz beim Papst (i.e., audience with the pope; pages 187–188)
- Menschen-Memory (i.e., human memory game; pages 59–60)
- Aus der Zeitung lesen (i.e., reading from the newspaper; pages 137–138)
- Briefe lesen (i.e., reading letters; pages 189–190)
- Texte rezitieren (i.e., reciting texts; pages 127–130)

#### Non-verbal communication—theory

Short theory units aimed at sensitizing the participants for non-verbal cues in daily life were an additional part of the training. Participants were encouraged to discuss their own attention to non-verbal signals in daily life (e.g., facial expressions, gestures, tone of voice) depending on the social context.

### Sudoku training

Sudoku (Japanese *s*$\dot{u}$doku, short for *s*$\dot{u}$ji wa dokushin ni kagiru, meaning “the numerals must remain single”) is a Japanese pastime where numbers have to be filled into a grid following certain rules. Typically, the grid has the width and the height of nine fields each resulting in an overall number of 81 fields. Some of the fields are prefilled with numbers ranging from 1 to 9. The player has to fill in the remaining fields adhering to the following rules: Every number between 1 and 9 has to be filled in exactly once in each row and each column of the grid. Moreover, each of these numbers has to appear exactly once in each of the nine 3-by-3 sub-grids of the main grid. The solution of each Sudoku puzzle can be found by logically applying the rules of the game. The difficulty is defined by the amount of prefilled fields and the numbers already given.

The SUDOKU training consisted of a four-week training program (19 days, 1 h per day). Participants were seated at single desks oriented in the same direction of view. Each participant received a pencil, several sheets of scratch papers and a copy of a Sudoku book (Rossa, 2009). This book includes different types of Sudoku puzzles and the degree of difficulty ranges from very easy (first chapter) to difficult (last chapter). Participants were instructed to solve the Sudoku puzzles one at a time. Participants were allowed to use the scratch papers to make notes, but the use of any other aids as well as talking to each other or copying from each other was prohibited. The training was monitored continuously by the examiner. Subsequent to each training session, the solved Sudoku puzzles were checked and errors were marked. In the following training session, Sudoku puzzles with errors had to be repeated before a new one could be started.

### Image acquisition

High resolution structural T1-weighted images [176 slices, slice thickness 1 mm, no gap, TR = 2300 ms, echo time (TE) = 2.96 ms, time to inversion (TI) = 1100 ms, voxel size: 1 × 1 × 1 mm^3^, field of view (FoV) = 256 × 256 mm^2^, magnetization prepared rapid acquisition gradient echo (MPRAGE) sequence] and functional images [30 axial slices acquired in an interleaved descending order, slice thickness 4 mm + 1 mm gap, TR = 1.7 s, TE = 30 ms, voxel size: 3 × 3 × 5 mm^3^, FoV = 192 × 192 mm^2^, echo-planar imaging (EPI) sequence] were recorded with a 3 T scanner (Siemens TIM TRIO, Erlangen, Germany). A field map [36 slices, slice thickness 3 mm + 1 mm gap, TR = 400 ms, TE(1) = 5.19 ms, TE(2) = 7.65 ms, voxel size: 3 × 3 × 4 mm^3^] was acquired to correct for image distortions.

### Analysis of behavioral data

Valence ratings and response times were treated as behavioral outcome variables. First, the valence ratings were transformed from symbolic to numerical values (– = 1, − = 2, + = 3, ++ = 4). Then mean absolute valence ratings for neutral and emotional (i.e., positive and negative) non-verbal cues were calculated on the above arbitrary scale where a value of 2.5 indicates “neutral” valence. These absolute valence ratings and the response times were then analyzed using IBM SPSS Statistics Version 19 (IBM Corp., Armonk, NY, USA) within the framework of a three-factorial ANOVA for repeated measure with non-verbal emotion (emotional, neutral) and time point (T0, T1) as within-subject factors and training type (NECT, SUDOKU) as between-subject factor. To clarify potential interactions between the participants' personality and the experimental factors, additional ANOVAs with the separate NEO-FFI personality factors as covariates were performed. For the correlation of individual training-associated changes in the behavioral sensitivity to non-verbal emotional cues with the respective cerebral activation patterns, the interaction term T0[EMO − NEU]−T1[EMO − NEU] was calculated from the valence ratings as well as from the response times of each participant.

### Analysis of FMRI data

Imaging data were analyzed with statistical parametric mapping software (SPM5, Wellcome Department of Imaging Neuroscience, London, UK). Preprocessing of the images comprised realignment, unwarping to correct for field distortions and to remove residual movement-related variance due to interactions between motion and field distortions (Andersson et al., 2001), coregistration with the anatomical data, normalization into MNI space (Montreal Neurological Institute, resampled voxel size: 3 × 3 × 3 mm^3^), and smoothing with a Gaussian filter [8 mm full width at half maximum (FWHM)]. The first five EPI images were discarded to exclude measurements preceding T1 equilibrium.

Responses to the stimuli of the main experiment were modeled separately for each trial as event-related responses employing a stick function time-locked to stimulus onset convolved with the hemodynamic responses function (HRF). For the face and voice localizer experiments, responses to the single categories (faces, houses, objects, and scenes in the face localizer and human voices, animal sounds, and environmental sounds in the voice localizer) were separately modeled using a box-car function corresponding to the duration of the respective blocks of stimuli convolved with the HRF. A high-pass filter with a cut-off frequency of 1/128 Hz was applied to reduce low-frequency components in the data. Serial autocorrelations within the data were accounted for by modeling the error term as an autoregressive process (Friston et al., 2002).

For the main experiment and the localizer experiments, data from the individual first-level general linear models were employed to create contrast images for each subject. These were then submitted to a second-level random-effect analysis to enable population inference.

In the main experiment, brain regions showing stronger responses during task performance than during rest were identified via the main contrast where all events where contrasted against the implicit resting baseline. Brain regions exhibiting stronger responses to non-verbal emotional stimuli (i.e., positively and negatively valenced expressions) than to non-verbal neutral stimuli were identified via the contrast EMO > NEU.

#### General and emotion-specific effects of NECT

To allow inference on general training effects for the processing of non-verbal expressions and differential training effects for the processing of non-verbal emotional and neutral expressions respectively, both of the above mentioned individual contrasts of interest were submitted to separate two-factorial whole-brain ANOVAs with time point (T0, T1) as within-subject factor and training type (NECT, SUDOKU) as between-subject factor. Results of the whole-brain analyses are reported at a height threshold of *p* < 0.001, uncorrected, and an extent threshold of *k* = 50 voxels corresponding to *p* < 0.05, family wise error (FWE) corrected for multiple comparisons across the whole-brain at the cluster level.

The source of any observed interaction effects was determined by applying one-sample *t*-tests to the mean parameter estimates extracted from the clusters with significant interaction effects for the contrast T0–T1 for both training types (NECT, SUDOKU).

#### Correlations of cerebral responses with personality (NEO-FFI)

In order to further investigate the relationship between the participants' personality traits and observed cerebral correlates of NECT, the mean contrast estimates were extracted from all brain regions with significant effects of NECT and correlated with the five NEO-FFI factors in the NECT group. To ascertain the specificity of potential correlations, the equivalent analysis was also performed in the SUDOKU group. All resulting *p* values are reported two-tailed and were Bonferoni-corrected for the number of NEO-FFI factors (*n* = 5).

#### Correlations of cerebral responses with NECT-associated behavioral changes in sensitivity to non-verbal emotional cues

As final add-on analyses, NECT-specific linear relationship of changes in the behavioral correlates of sensitivity to non-verbal emotional cues [i.e., the following contrast in valence ratings and response times: NECT_T0_[EMO − NEU] − T1[EMO − NEU] vs. SUDOKU_T0_[EMO − NEU] − T1[EMO − NEU]] were tested by performing a correlation analysis between the respective behavioral and cerebral activation contrasts at the population level. Statistical analyses were performed with a threshold of *p* < 0.001, uncorrected, at voxel level for whole-brain analyses. Results were FWE-corrected at cluster level (*p* < 0.05). A more sensitive threshold of *p* < 0.01, uncorrected at voxel level, was used for regions of interest (ROI) analyses within face- and voice-selective regions as well as within regions with general NECT-associated changes in cerebral activation. Here, small volume correction (SVC; Worsley et al., 1996) for the size of the respective ROI together with FWE correction (*p* < 0.05) at cluster level was applied. Observed effects were post-hoc further investigated by extracting the mean contrast estimates from significant clusters for both training groups (NECT, SUDOKU) and performing separate bivariate correlation analyses (Pearson).

#### Functional localizer experiments for voice- and face-selective brain regions

For the face localizer experiment, the responses to faces were contrasted against the responses to houses, objects, and natural scenes, while for the voice localizer experiment responses to human voices were contrasted against animal and environmental sounds.

Results of the whole-brain group analyses are reported at a height threshold of *p* < 0.0001, uncorrected, and an extent threshold of *k* = 10 voxels corresponding to *p* < 0.05, FWE corrected for multiple comparisons across the whole-brain at the cluster level. The strict statistical threshold at the voxel level allows a non-overlapping localization of face- and voice-selective brain areas. Face- and voice-selective regions were then used as ROI for additional analyses of NECT effects with the contrasts described above using SVC and FWE-correction.

The Automated Anatomic Labeling (AAL) toolbox implemented in SPM (Tzourio-Mazoyer et al., 2002) was used for the anatomic cluster labeling.

### Analysis of structural MRI data

The goal of the voxel based morphometry analysis was to identify brain regions with changes in GM volume associated with NECT. The voxel based morphometry toolbox (VBM8) implemented in the SPM8 software environment was used for the preprocessing of the high resolution structural T1 images. Preprocessing was performed using the VBM8 default pipeline recommended for longitudinal data and included realignment, bias correction, segmentation into GM, white matter and cerebrospinal fluid and normalization. The voxels were resized to 1.5 × 1.5 × 1.5 mm^3^ during preprocessing. VBM8 by default produces modulated images. This means that the voxel values of the GM images are multiplied by the non-linear components of the normalization procedure thus correcting the data for individual brain sizes before the setup of a statistical model and enabling the analysis of relative differences in regional GM volume. The GM images were smoothed with an 8-mm FWHM Gaussian kernel and then analyzed within the framework of a two factorial ANOVA with time point as within-subject factor and training type as between-subject factor. The interaction term between time point and training type was defined as contrast of interest. Primary ROIs for the VBM analysis were those regions with a significant effect of NECT at the level of cerebral activation (fMRI) and face- as well as voice-selective brain regions. These analyses were complemented with a whole-brain analysis to rule out unspecific NECT associated structural alterations. Results are reported at a height threshold of *p* < 0.001, uncorrected, and *p* < 0.05 with SVC for the respective ROI using FWE-correction at cluster level. For the whole-brain analysis FWE-correction at cluster level was used in the same fashion. For areas with a significant interaction between time point and training type, mean GM values were extracted and results validated by testing the time point by training type interaction term after correcting for differences in GM volume at T0.

## Results

### Behavioral responses

#### Valence ratings

**Figure 1:**
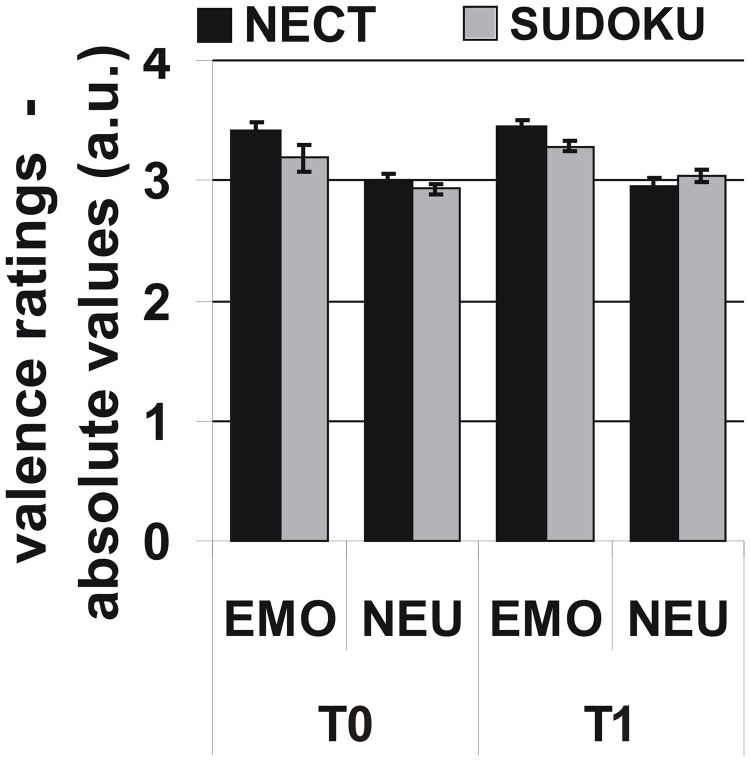
**Behavioral responses: More extreme absolute valence ratings for emotional than for neutral stimuli, but no systematic influence of training type or time point on valence ratings or respective interactions**. Note: Initial rating scale: [– − + ++] transformed to numeric values (1–4). Neutral = 2.5; absolute valence ratings relative to “neutral” are given for emotional stimuli.

The 2 × 2 × 2-factorial ANOVA revealed that emotional stimuli received more extreme valence ratings than neutral stimuli [*F*_(1, 12)_ = 35.6, *p* < 0.001; see Figure 1]. However, no significant effects were observed for the factors time point, training type, or the interactions between the three experimental factors [all *F*_(1, 12)_ ≤ 3.0, all *p* ≥ 0.11]. Additional ANOVAs with the separate NEO-FFI personality factors as covariates did not yield any significant interactions with any of the experimental factors [all *F*_(1, 12)_ = 1.5, all *p* = 0.24].

#### Response times

Responses to emotional stimuli were faster than to neutral stimuli [*F*_(1, 12)_ = 38.8, *p* < 0.001; emotional stimuli: mean ± *SEM*: 1639 ± 32 ms, neutral stimuli: 1800 ± 42 ms]. For the remaining factors time point, training type, or the interactions between all three experimental factors no significant effects emerged [all *F*_(1, 12)_ ≤ 3.4, all *p* ≥ 0.09]. Furthermore, no significant interactions between the NEO-FFI factors and any of the experimental factors were observed [all *F*_(1, 12)_ ≤ 4.3, all *p* ≥ 0.06].

### Cerebral responses

#### General and emotion-specific effects of NECT

**Figure 2:**
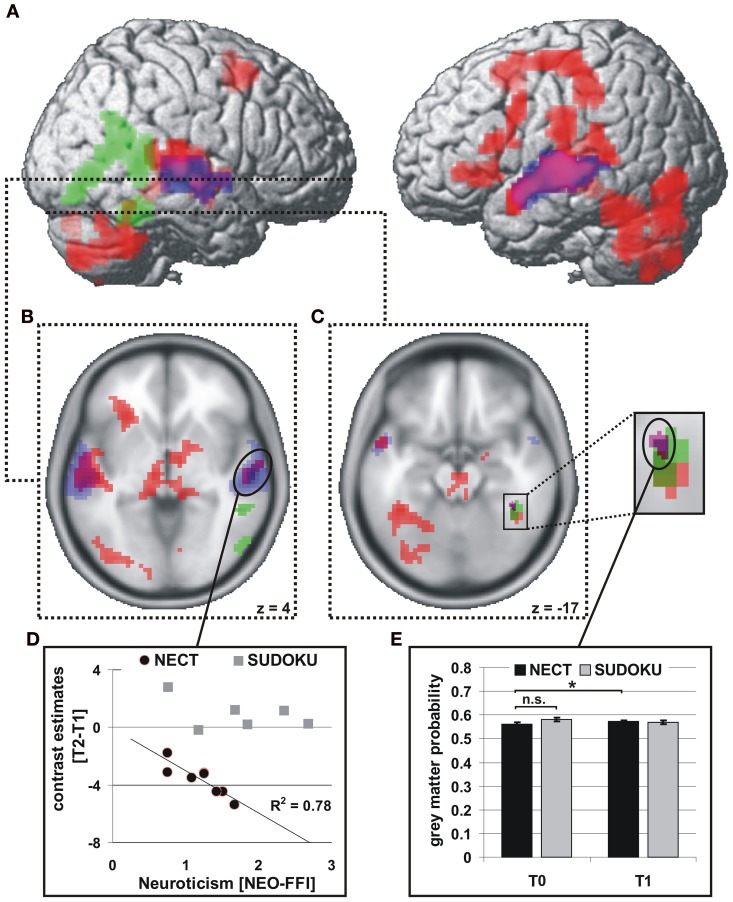
**Brain regions with significant functional and structural effects of NECT**. fMRI: interaction of training type with time point of measurement (red; **A–C**), *p* < 0.001, uncorrected, *k* > 47 voxels, corresponding to *p* < 0.05, FWE corrected at cluster level. Voice-selective areas (blue) and face-selective areas (green), *p* < 0.0001, uncorrected, *p* < 0.05 FWE whole-brain-corrected at cluster level. **(D)** Linear relationship between training-associated changes in neural activity and neuroticism in the NECT-group in the right mSTG. VBM: **(E)** interaction of training type with time point of measurement in the right FFA (purple; **C**), *p* < 0.001, uncorrected, *p* < 0.05 small-volume FWE corrected (right FFA) with a significant increase in NECT group. ^*^p < 0.05.

NECT-specific reductions in brain activity were revealed as an interaction between time point and training type within the framework of a whole-brain ANOVA in a distributed set of brain regions including face- and voice-selective areas of the visual and auditory cortices, STS, inferior frontal cortex, motor-related regions and cerebellum (see Figures 2A–C; Tables 1, 2). Post-hoc analysis of the mean parameter estimates from the clusters with significant interaction effects revealed that the interaction was uniformly due to a NECT-associated decrease in cerebral activation (all *T* ≥ 5.5, all *p* ≤ 0.001) while no significant change in the cerebral activation patterns occurred in the SUDOKU group (all *T* ≤ 2.2, *p* ≥ 0.08). An additional investigation of NECT effects specific for emotional non-verbal signals as compared to neutral non-verbal stimuli, framed as a second order interaction between time point, training type and non-verbal emotional stimulus content (i.e., emotional vs. neutral), did not yield any whole-brain significant results. Applying a more sensitive threshold of *p* < 0.01 uncorrected, however, demonstrated several clusters in inferolateral and dorsolateral regions of the prefrontal cortex as well as the anterior and middle cingulum extending into the posterior cingulum and precuneus (see Table 3). Please note that these results are only reported for the purpose of completeness and are purely descriptive.

**Table 1:** **Brain areas with training (NECT) specific changes in their responses to non-verbal cues from voice and face as determined by the whole-brain interaction analysis between time point (T0, T1) and training type (NECT, SUDOKU)**.

	***x***	***y***	***z***	***Z*-score (peak voxel)**	**Cluster size (voxel)**
L superior and middle temporal gyri/ L postcentral gyrus/ L Rolandic operculum/ L supramarginal gyrus/ L Heschl's gyrus ***[left STS]***	−48	−18	6	5.3	486
R+L cerebelum/ L fusiform gyrus/ L inferior and middle occipital gyri/ L inferior temporal gyrus	9	−78	−45	4.6	833
Midbrain/ L+R thalamus/ R+L lingual gyri/ R cerebellum/ L+R parahippocampal gyrus/ R hippocampus ***[midbrain/thalamus]***	6	−15	−12	4.5	359
L+R supplementary motor area/ L precentral gyrus/ L postcentral gyrus/ L inferior parietal gyrus/ L superior and middlefrontal gyri	−3	9	48	4.5	294
R superior and middle temporal gyri/ R Heschl's gyrus ***[right middle/posterior STG]***	57	−24	3	4.3	146
L insula/ L inferior frontal gyrus, partes orbitalis and triangularis	−27	27	−3	4.1	68
L inferior frontal gyrus, partes opercularis and triangularis/ L precentral gyrus	−39	15	15	4.0	72
R+L cerebellum/ R fusiform gyrus	33	−48	−39	3.8	74

Activations thresholded at p < 0.001, uncorrected with a cluster size k ≥ 50, corresponding to p < 0.05, FWE corrected for multiple comparisons across the whole-brain. Voxel size 3 × 3 × 3 mm*^3^*. The anatomical descriptions in brackets represent short descriptions of the respective clusters. The clusters are referred to using these descriptions in the text.

**Table 2:** **Voice- and face-selective brain areas with training (NECT) specific changes in their responses to non-verbal cues from voice and face**.

	***x***	***y***	***z***	***Z*-score (peak voxel)**	**Cluster size (voxel)**
ROI: R temporal voice area (TVA)	60	−18	−3	6.5	237
Effect of NECT within R TVA: R superior temporal gyrus	57	−24	3	4.3	41
ROI: L temporal voice area	−54	−24	0	5.4	346
Effect of NECT within L TVA: L superior and middle temporal gyrus	−51	−21	−3	5.0	177
ROI: R fusiform face area	42	−45	−21	5.0	45
Effect of NECT within R FFA: R fusiform gyrus	42	−51	−21	3.4	8
ROI: R STS face area	48	−39	6	5.1	272
Effect of NECT within R STS-FA: R superior and middle temporal gyrus	45	−36	12	3.8	11

Localizer experiment activations are thresholded at p < 0.0001, uncorrected, with a cluster size k ≥ 10 (face localizer) corresponding to p < 0.05, FWE corrected for multiple comparisons across the whole-brain. Within-ROI NECT effects are thresholded at p < 0.001, uncorrected. All within-ROI cluster NECT-effects are significant with p < 0.05, FWE corrected for multiple comparisons across the respective ROI (SVC). Voxel size 3 × 3 × 3 mm*^3^*.

**Table 3:** **Brain areas with differential training (NECT) specific changes in their responses to emotional and neutral non-verbal cues from voice and face**.

	***x***	***y***	***z***	***Z*-score (peak voxel)**	**Cluster size (voxel)**
R inferior frontal gyrus, partes triangularis, orbitalis and opercularis	57	24	9	3.7	92
R middle frontal gyrus/ R precentral gyrus	45	9	42	3.5	56
L calcarine gyrus/ L precuneus	−6	−48	6	3.4	41
R inferior and middle frontal gyri, partes orbitals	39	36	−9	3.3	45
L+R anterior cingulum/ L+R superior frontal gyrus, pars medialis	3	45	12	3.3	41
R+L middle cingulum/ R precuneus/ L posterior cingulum	6	−27	36	3.2	121
R+L thalamus	6	−18	15	3.2	53

Activations thresholded at p < 0.01, uncorrected with a cluster size k ≥ 40. Voxel size 3 × 3 × 3 mm^3^. Reported for descriptive purposes only.

#### Correlations of cerebral responses with personality (NEO-FFI)

There were no significant differences with regard to the separate NEO-FFI personality factors or age between the training groups [all abs(*t*_(12)_) ≤ 1.9, all *p* ≥ 0.09].

Of the five personality factors investigated, solely neuroticism exhibited a significant linear relationship with the size of NECT-associated decreases in cerebral activation during processing of non-verbal cues from voice and face in three regions, namely the right middle/posterior superior temporal gyrus (STG; *r* = −0.88, *p* = 0.004; see Figures 2B,D), the left STS (*r* = −0.78, *p* = 0.024; see Figure 2B) and the midbrain/thalamus (*r* = −0.71, *p* = 0.048; see Figure 2B). Only the linear relationship in the right middle/posterior STG survived correction for the number of personality factors tested (*p* = 0.018). No equivalent significant linear relationships between the effects of SUDOKU training and any of the personality factors were observed.

#### Correlations of cerebral responses with NECT-associated behavioral changes in sensitivity to non-verbal emotional cues

**Figure 3:**
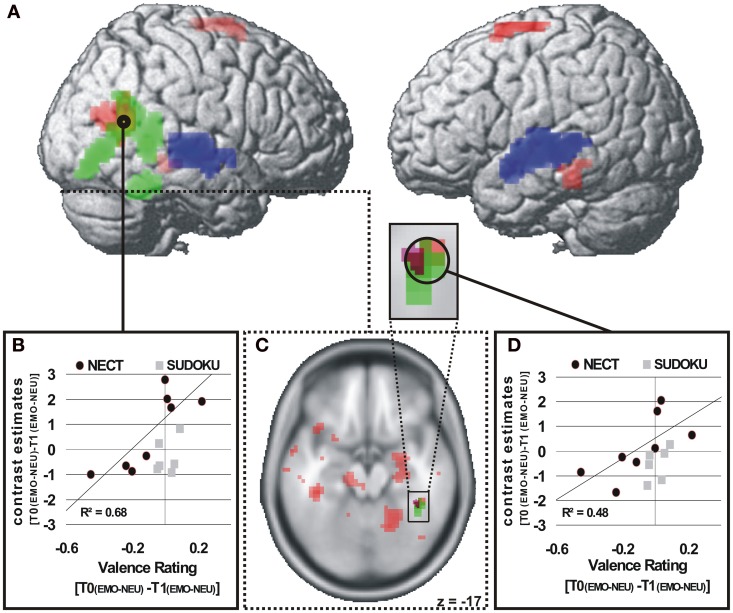
**Brain regions with a NECT-specific association between changes in behavioral ratings and cerebral responses (A,C)**. *p* < 0.01 **(A)**, *p* < 0.05 **(B)**, uncorrected, *k* ≥ 20 voxels **(A)**. Significant positive association of changes in behavioral emotion-sensitivity (T0[EMO - NEU] - T1[EMO - NEU]) and changes in cerebral responses in the NECT group and a non-significant association in the SUDOKU group in the right STS face area **(A,B)** and in the right FFA **(C,D)**. Voice-selective areas (blue) and face-selective areas (green), *p* < 0.0001, uncorrected, *p* < 0.05 FWE whole-brain-corrected at cluster level.

***Valence ratings.*** The ROI analysis of the voice- and face-selective brain regions and brain regions with significant NECT-effects revealed that the right STS face area (STS-FA; coordinates of peak voxel: 48x, −57y, 15z; *Z* value of peak voxel: 3.7; cluster size: 34 voxels; see Figures 3A,B) and the right FFA (coordinates of peak voxel: 42x, −42y, −24z; *Z* value of peak voxel: 3.0; cluster size: 2 voxels; see Figures 3A,C,D) exhibited a positive linear relationship between NECT-associated changes in sensitivity to emotional non-verbal cues and NECT-associated increases in sensitivity to such cues. At the whole-brain level no such relationship was observed.

**Figure 4:**
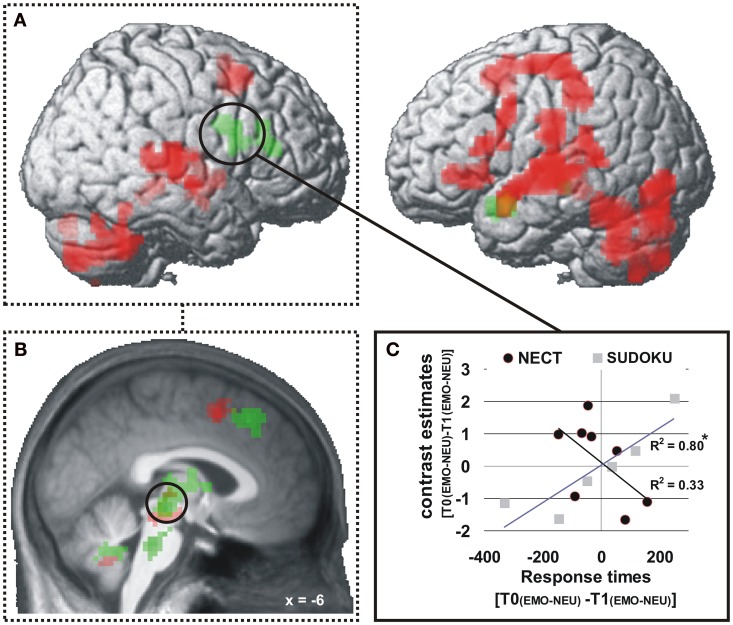
**Brain regions with training (NECT vs. SUDOKU) specific associations between changes in response times and cerebral responses (A,B)**. *p* < 0.001 **(A)**, *p* < 0.01 **(B)**, uncorrected, *k* ≥ 20 voxels **(A)**. A significant negative association of changes in behavioral emotion-sensitivity (as measured by the response times contrast: T0[EMO − NEU] − T1[EMO − NEU]; nota bene: due to generally longer response times to neutral than to emotional cues a positive value of this interaction terms corresponds to an increase in emotion-sensitivity) and changes in cerebral responses in the SUDOKU group is reversed in the NECT group **(C)**; here exemplified based on extracted mean contrast estimates from the whole-brain significant cluster in the right inferior frontal gyrus. ^*^p < 0.05.

***Response times.*** At the whole-brain level significant training specific (i.e., NECT vs. SUDOKU) associations between changes in the behavioral emotion-sensitivity and cerebral responses were observed in the left temporal pole and the right inferior frontal gyrus (see Table 4 and Figure 4A). In a ROI analysis of the voice- and face-selective brain regions and brain regions with significant NECT-effects with a more sensitive voxel-wise threshold of *p* < 0.01, this effect was also found in the midbrain/thalamus ROI (coordinates of peak voxel: −6x, −24y, −6z; Z value of peak voxel: 3.9; cluster size: 65 voxels; see Figure 4B). Post-hoc analyses revealed that the observed effects were uniformly driven by the difference between a significant negative linear relationship between the changes in the response time correlate of emotion sensitivity T0[EMO − NEU] −T1[EMO − NEU] and its cerebral counterpart in the SUDOKU group [*r*_(6)_ ≥ 0.89, *p* ≤ 0.02], on the one hand, and the reversed (i.e., positive) linear relationship in the NECT group (see Figure 4C). This relationship in the NECT group was significant in the left temporal pole [*r*_(4)_ = 0.72; *p* = 0.046] and non-significant in the right inferior frontal gyrus [*r*_(4)_ = 0.58, *p* = 0.13] and the midbrain/thalamus ROI [*r*_(4)_ = 0.45, *p* = 0.26]. In other words, an increase in the response time difference between non-verbal emotional and neutral cues was accompanied by an increase in activation differences between these types of cues in the NECT group. In the SUDOKU group, the same behavioral pattern was associated with decreased activation differences between emotional and neutral cues.

**Table 4:** **Brain areas with training (NECT vs. SUDOKU) specific associations between training-induced changes in the behavioral sensitivity to non-verbal emotional cues as measured by response times and the respective cerebral activation patterns (contrast of interest: T0[EMO - NEU]-T1[EMO - NEU])**.

	***x***	***y***	***z***	***Z*-score (peak voxel)**	**Cluster size (voxel)**
L superior temporal pole	−39	9	−18	4.28	47*
Midbrain	−3	−24	−9	4.25	24
R inferior frontal gyrus, partes opercularis and triangularis	36	9	24	3.95	49*
R inferior frontal gyrus, pars triangularis	48	27	27	3.77	20

Activations thresholded at p < 0.001, uncorrected, with a minimal cluster size of k ≥ 20 voxels. Activations with a cluster size k ≥ 46, correspond to a significance level of p < 0.05, FWE corrected for multiple comparisons across the whole-brain and are marked with an asterisk. Voxel size 3 × 3 × 3 mm^3^.

### NECT-associated changes in gray matter volume

Only in the right FFA an interaction between time point and training type was observed with regard to GM volume (coordinates of peak voxel: 40x, −42y, −18z; *Z* value of peak voxel: 3.5; cluster size: 24 voxels; see Figures 2C,E). Neither in the other face- or voice-selective areas or in the brain regions with NECT-associated activation decreases nor at the whole-brain level such an effect could be observed. The interaction effect in the right FFA was driven by a significant increase in GM volume in the NECT group [*t*_(7) = 2.2_, *p* = 0.03] while there were no differences between the two training groups at T0 [*t*_(12)_ = 1.5, *p* = 0.148]. After removal of variance in GM volume at T0, the NECT-associated increase in GM volume remained significant [*t*_(7)_ = 3.0, *p* = 0.005].

## Discussion

This is, to our knowledge, the first combined fMRI and structural cerebral imaging study, reporting the specific effects of a four-week-long NECT in healthy individuals. Our results afford a first view on the neuronal underpinnings of non-verbal trainings which—implemented in various forms—have been demonstrated to be effective in enhancing non-verbal decoding skills and sensitivity (e.g., Klinzing and Jackson, 1987; Constanzo, 1992; Matsumoto and Hwang, 2011).

### General effects of NECT on cerebral activation patterns

In accordance with our expectations, NECT-specific alterations in brain activity were observed in a distributed set of brain regions including face- and voice-selective areas of the visual and auditory cortex, the STS, inferior frontal cortex, insula and thalamus which have all been demonstrated to be involved in the processing of audiovisual non-verbal emotional signals (for reviews see Campanella and Belin, 2007; Brück et al., 2011b; Kreifelts et al., 2013). Moreover, NECT induced altered activation patterns in motor-related regions, the cerebellum and the parietal cortex. All brain regions with NECT-specific activation alterations showed a uniform pattern with decreased activation during the perception and evaluation of non-verbal signals from voice and face which fit in well with earlier reports of decreased cerebral activation after procedural (Friston et al., 1992; Steele and Penhune, 2010) and perceptual (Schiltz et al., 1999) learning. These decreases may constitute a correlate of training-induced familiarization with the evaluation of face/voice stimuli including less effortful perceptual processing as well as a facilitation of stimulus-directed attention, decision making and response selection. It is worth noting, that the observed decreases in activation appear similar to the effect of so called “repetition suppression,” a phenomenon occurring after the repeated presentation of identical stimuli. Repetition suppression effects presumably reflect bottom-up sharpening of neural responses (Larsson and Smith, 2012) but also top-down mediated perceptual expectations (Summerfield et al., 2008). Although, in the present study, “simple” repetition suppression can be ruled out as source of the observed effects due to the fact that each stimulus was shown exactly twice during the course of the study in both training groups, nevertheless NECT might tap into similar bottom-up and top-down neuronal tuning processes, not via the repetition of identical stimuli but via similarities between the tasks and stimuli of the training and during the fMRI experiment.

Decreases of cerebral responses through NECT in healthy individuals also stand in contrast to the observation of cerebral activation increases observed in schizophrenic patients after an emotion recognition training (Habel et al., 2010). Given the paucity of data with regard to the neural correlates of such non-verbal trainings, further studies are needed which directly compare training effects in psychiatric populations and healthy individuals as an active control condition to determine if there are indeed fundamental differences in the neural correlates of non-verbal emotional trainings between these groups.

With regard to differential NECT training effects for emotional and neutral non-verbal signals, the results of our study were negative: There was a non-significant tendency toward greater absolute valence rating differences between emotional and neutral stimuli after NECT at the behavioral level (see Figure 1) and several brain areas in the right orbitofrontal cortex, right lateral prefrontal cortex as well as areas in the anterior and middle cingulate cortex extending into the precuneus where differential NECT training effects for emotional and neutral non-verbal stimuli failed whole-brain significance. Two potential reasons for this negative finding should be considered: Firstly, the negative finding might be due to a lack of power. This notion is supported by the suggestive correspondence between the behavioral tendency toward a more sensitive discrimination of emotional and neutral stimuli and sub-threshold cerebral activation patterns occurring in brain areas previously associated with the evaluation of emotional stimuli, mentalizing (i.e., inferences on the mental states and intentions), and supramodal emotion processing (Amodio and Frith, 2006; Brück et al., 2011b; Klasen et al., 2011, 2012).

A second reason might be that in the context of a valence rating task “neutral” and “emotional” stimuli are processed similarly as both types of stimuli are evaluated with respect to their emotional valence. While offering a direct behavioral correlate of emotional evaluation, this experimental context may have decreased activation differences for “emotional” and “neutral” stimuli relative to, for example, an implicit emotion processing design with a gender discrimination task as demonstrated in previous studies (Hariri et al., 2000; Lange et al., 2003).

### Personality-dependent neuronal effects of NECT

The middle and posterior aspects of the right STG, and to a lesser degree also the left STS and posterior thalamus/midbrain exhibited a specific characteristic in that neuronal NECT effects here were strongly and positively correlated with the personality trait of neuroticism. In light of the known association between neuroticism and depression as well as anxiety disorders (Brandes and Bienvenu, 2006; Klein et al., 2011) with high levels of neuroticism in anxious and depressed patient samples, this association may appear relevant for future studies with respect to the strength of observable neuronal NECT effects in clinical samples of anxious and depressed patients. Here, the present data set suggests that the present experimental design may be even more sensitive in detecting cerebral correlates of NECT in clinical groups with strong neurotic personality traits.

### NECT induced changes in sensitivity to emotional signals: correlations between behavior and cerebral activation

For both, the valence rating correlate as well as the response time correlate of changes in emotion sensitivity, corresponding cerebral activation patterns were observed:

#### Valence ratings

Exclusively within the face-selective areas in the right pSTS and the right fusiform gyrus a NECT-specific correlation between changes in the behavioral sensitivity to non-verbal emotional signals from voice and face and corresponding activation patterns was observed. The degree to which individuals after NECT perceived greater absolute valence differences between neutral and emotional stimuli was correlated with an increase in cerebral sensitivity to emotional as compared to neutral non-verbal stimuli. The exclusive observation of such associations in face-selective cortices supports the hypothesis that sensory tuning in the decoding of facial expressions lies at the neural basis of NECT-induced behavioral alterations in sensitivity to non-verbal emotional cues. It remains an open question if the lack of such a correlation in the voice-selective sensory cortices is the result of an innate preference of humans to base the evaluation of social signals on visual stimulus components (e.g., DePaulo et al., 1978), or if face-selective sensory cortices are more training sensitive in the plasticity of their neuronal responses than voice-selective areas. Thirdly, despite the audiovisual nature of NECT, a potential tendency of the participants toward the use of visual non-verbal signals during NECT might explain the observed patterns of association between behavioral data and cerebral activation. Moreover, the observation of correlations between emotion sensitivity and cerebral activation patterns both in the STS-FA and FFA, justifies to very cautiously argue against the view of a strict functional segregation of these two face-processing modules (Haxby et al., 2000) where the FFA processes invariant aspects of faces (e.g., gender or identity) and the STS-FA processes dynamic aspects of faces (e.g., facial expressions or gaze).

Finally, the largest cortical cluster with a correlation between training induced increases in facial emotion recognition in schizophrenic patients and corresponding increases in cerebral responses (Habel et al., 2010) was observed in the pSTS in a very similar location as the association between behavioral and cerebral correlates of NECT. This points to the STS-FA as a central cortical module linking behavioral and neuronal effects of non-verbal emotional recognition and communication trainings.

#### Response times

For changes in the response time-based behavioral measure of emotion sensitivity, neural activation correlates were revealed in the left temporal pole, the right inferior frontal gyrus and midbrain/thalamus. While individually increased behavioral sensitivity to emotional cues was correlated with a decrease in cerebral sensitivity to these cues in the SUDOKU group, NECT led to the reversed pattern with parallel changes in behavioral and cerebral correlates of emotion sensitivity.

Parallel to the association of valence rating changes and cerebral activation patterns, interpretations of the above findings in a rather small study like the present one have to be phrased with caution and need to be treated as preliminary.

The most striking feature of the analyses presented here is that NECT induced a qualitative change in the association of behavioral and cerebral correlates of emotion-sensitivity. This finding points to the left temporal pole, the right inferior frontal gyrus and the midbrain/thalamus as cerebral structures which instantiate NECT-driven training effects as interfaces between the evaluation of non-verbal signals and the selection of responses to these signals. The assumption that the right inferior frontal gyrus is implicated in this process is in line with findings that the lateral prefrontal cortex is activated during the evaluation of non-verbal emotional stimulus content (e.g., Schirmer and Kotz, 2006; Brück et al., 2011b; Ethofer et al., 2013) and structurally connected to voice- and face-selective cortices, the face/voice integration region of the STS as well as the supplementary motor area (Ethofer et al., 2013). Also, the temporal pole has been implicated in emotion processing (Olson et al., 2007) and multimodal integration of audiovisual emotional signal (Kreifelts et al., 2007) although its specific functional properties remain at least partially unresolved. Olson et al. (2007) hypothesized that the temporal pole binds complex, perceptual inputs to visceral emotional responses. Present results suggest an additional role of the temporal pole in binding complex emotional percepts to voluntary motor responses. As for the midbrain/thalamus cluster, a comparison with the activation coordinates of previous studies on audiovisual integration and supramodal representation of emotional signals (Kreifelts et al., 2007, 2010; Klasen et al., 2011) supports the hypothesis that the location of this cluster overlaps with a brain region in the posterior thalamus which has been demonstrated not only to be involved in the audiovisual integration of emotional signals (Kreifelts et al., 2007, 2010; Klasen et al., 2011) but also to exhibit a linear relationship of hemodynamic with behavioral responses (i.e., emotion recognition rates; Kreifelts et al., 2007).

### NECT-induced structural plasticity

The increase in GM volume after NECT in the right FFA represents, to our knowledge, the first demonstration of specific structural plasticity induced by a complex emotional communication training. These results fit in well with the growing body of studies investigating the structural effects of various motor-related trainings (Driemeyer et al., 2008; Taubert et al., 2010; Granert et al., 2011) but also visual perceptual training (Ditye et al., 2013). These concordantly indicate dynamic increases in GM volume in areas functionally associated with the trained tasks. Regarded from this perspective, the present structural findings argue for a pivotal position of the right FFA within the cerebral network exhibiting dynamic functional and structural alterations as neuronal correlates of NECT. Nevertheless, one may ask if the small size of the FFA as a functional cortical module does not somewhat boost the sensitivity to detect even smaller effects.

### Limitations and perspectives

A limitation on the implications of the present study is its small sample size which allows only large (i.e., with regard to effect size) effects of NECT to be discovered. Therefore, a training study with a larger sample size may depict a more complex and fine-grained pattern of cerebral alterations induced by NECT. Nevertheless, the considerable strength of the observed effects affords a first impression of NECT-induced functional and structural cerebral alterations and may serve as a good starting point for future studies including larger samples of healthy individuals and patients with psychiatric conditions. A second important point pertains specifically to the neurofunctional correlates of NECT: In the present study these are inherently tied to the cognitive context of a valence rating task. Depending on the psychiatric conditions investigated in future studies, it would be sensible to accommodate disorder-specific neuropsychological and behavioral alterations in the processing of non-verbal emotional signals within the experimental setup (i.e., both stimulus material and task) in order to capture an optimized estimate of disease-relevant behavioral alterations and their neuronal correlates.

## Conclusion

Here, we demonstrated in healthy participants, an association of NECT-induced changes in brain function and structure with changes in the evaluation of non-verbal emotional stimulus content and neuroticism. Based on these findings we conclude that the present experimental design may be a very valuable neuroimaging probe not only for the investigation of the neural bases of altered processing of non-verbal emotional cues in psychiatric disorders but also for the assessment of the influence of therapeutic interventions on brain function and structure.

### Conflict of interest statement

The authors declare that the research was conducted in the absence of any commercial or financial relationships that could be construed as a potential conflict of interest.
